# Do Capuchin Monkeys (*Cebus apella*) Diagnose Causal Relations in the Absence of a Direct Reward?

**DOI:** 10.1371/journal.pone.0088595

**Published:** 2014-02-19

**Authors:** Brian J. Edwards, Benjamin M. Rottman, Maya Shankar, Riana Betzler, Vladimir Chituc, Ricardo Rodriguez, Liara Silva, Leah Wibecan, Jane Widness, Laurie R. Santos

**Affiliations:** 1 Department of Psychology, Northwestern University, Evanston, Illinois, United States of America; 2 Yale University, New Haven, Connecticut, United States of America; Durham University, United Kingdom

## Abstract

We adapted a method from developmental psychology [1] to explore whether capuchin monkeys (*Cebus apella*) would place objects on a “blicket detector” machine to diagnose causal relations in the absence of a direct reward. Across five experiments, monkeys could place different objects on the machine and obtain evidence about the objects’ causal properties based on whether each object “activated” the machine. In Experiments 1–3, monkeys received both audiovisual cues and a food reward whenever the machine activated. In these experiments, monkeys spontaneously placed objects on the machine and succeeded at discriminating various patterns of statistical evidence. In Experiments 4 and 5, we modified the procedure so that in the learning trials, monkeys received the audiovisual cues when the machine activated, but did not receive a food reward. In these experiments, monkeys failed to test novel objects in the absence of an immediate food reward, even when doing so could provide critical information about how to obtain a reward in future test trials in which the food reward delivery device was reattached. The present studies suggest that the gap between human and animal causal cognition may be in part a gap of motivation. Specifically, we propose that monkey causal learning is motivated by the desire to obtain a direct reward, and that unlike humans, monkeys do not engage in learning for learning’s sake.

## Introduction

Human causal cognition is impressive not only because we excel at learning causal relationships that are present in our environment but also because we actively search for causal explanations and attempt to diagnose how causal systems work (see reviews in [2], [3]). Indeed, the search for explanation is so ubiquitous in everyday life that we are often motivated to learn about causal structures even in the absence of direct benefits. For example, many people are curious about how the immune system fights disease and how automobiles work, and not just when they are sick or when their car will not start.

Recent work in developmental psychology suggests that our understanding of and curiosity about causal relations seems to develop within the first few years of life (see reviews in [4], [5]). Schulz and Bonawitz [6], for example, examined children’s ability to learn causal relations in the absence of a direct reward through their exploratory play. They presented preschoolers with different types of evidence concerning how a toy box worked. In one condition, children received confounded evidence. They saw that rotating two levers on the toy box simultaneously caused two different objects to pop out from inside. Based on this presentation, it was unclear whether one lever controlled both objects or whether each lever controlled a different object, and if so, which lever controlled which object. In another condition, children received unconfounded evidence. In addition to seeing the effects of both levers rotated simultaneously, children in this condition also saw the effects of rotating each lever separately, which provided them with full causal information about how the toy worked. After receiving either confounded or unconfounded evidence, children were allowed to play freely with the familiar toy from the first part of the study and with a novel toy. Children in the unconfounded evidence condition preferred to play with the novel toy, suggesting that they were less interested in the original familiar toy after receiving the evidence of how it worked. Children in the confounded evidence condition, in contrast, showed a greater tendency to play with the familiar toy, with many of these children performing actions to diagnose the toy’s causal structure. Schulz and Bonawitz [6] interpreted this pattern of results as evidence that children who received only confounded evidence about the toy remained curious about how that toy worked and thus continued playing with it accordingly.

Work using similar paradigms has shown that children presented with confounded evidence played with a toy in a more exploratory manner compared to children presented with unconfounded evidence, who played more exploitatively [7], [8]. Additionally, Legare [9] found that children proposing different explanations for anomalous data engaged in different patterns of exploratory play. Taken together, these results suggest that like adults, children may be motivated to diagnose causal structures even in situations in which such diagnoses are not immediately relevant or instrumentally beneficial.

In contrast to the strong evidence that children perform diagnostic actions to learn causal relations, there is little consensus regarding whether other animals possess the kinds of learning mechanisms that children use to acquire causal knowledge (see reviews in [10], [11]). Traditionally, most researchers have assumed that animals’ causal reasoning can be reduced to simple associative learning mechanisms (e.g., [12]; see also [13]–[16] for reviews). More recent work, however, suggests that in at least some respects, animals may be capable of higher-level causal reasoning similar to that of humans.

Blaisdell et al. [17], for example, tested whether rats are capable of predicting the results of interventions based on observational learning, a capacity that is a prominent feature of Bayes net theory, but that is irreducible to associative learning (e.g., [18]). They introduced rats to a causal system in which a light cue (*L*) was followed by a tone (*T*), and separately, the light cue (*L*) was followed by food (*F*). That is, the light cue appeared to be a common cause of the tone and food; *T* ← *L* → *F*. In the test phase, participants were either able to produce the tone by pressing a lever (condition intervene-*T*) or they observed the tone independently of pressing the lever (condition observe-*T*). Blaisdell et al. assessed rats’ expectations of food given that *T* was present by measuring the frequency of nose pokes into the food dispenser and found that rats in the intervene-*T* condition had a weaker expectation of food than rats in the observe-*T* condition, an inference that is consistent with a Bayes net account, but not associative accounts of animal causal learning (but see [11], [19] for critiques and alternative interpretations).

Although the present work is not motivated by the debate regarding whether animal cognition is reducible to associative learning mechanisms, this literature is relevant in that associative learning theories predict that animal learning is solely geared towards obtaining rewards [20]. This view suggests that animals act randomly in their environment until they engage in behavior that produces a reward. The reward reinforces the associated behavior, increasing its frequency. As we have reviewed earlier, human children engage in *diagnostic* behavior to learn how causal systems work. In this paper, we ask whether animals also perform diagnostic actions to learn about causal relations in their environment.

In one study of whether animals engage in diagnostic causal reasoning [21], chimpanzees (*Pan troglodytes*) were trained to place blocks upright, receiving a food reward for performing this task successfully. On some trials, chimpanzees were presented with a “sham block” that could not be placed upright. In contrast to human children tested on an analogous task, chimpanzees failed to inspect the sham block in trials in which there was no visual difference between the sham blocks and functional blocks. These results suggest that even our closest living animal relative fails to engage in diagnostic reasoning at the same level as young human children. However, despite the absence of evidence (and perhaps evidence of an absence) of diagnostic behavior in non-human primates, chimpanzees and other non-human primates can engage in sophisticated tool-use and object manipulation behaviors [22], [23], and can use physical cues (e.g., whether a cup makes a noise when shaken) to infer whether a container is baited with food [24]–[26].

Although relatively little work has tested animals’ diagnostic reasoning directly, there is a growing body of work examining whether animals– particularly non-human primates– possess metacognitive abilities to seek out more information when it’s needed. More specifically, this work has explored whether primates have the capacity to monitor the state of their own knowledge (see review in [27]). Although the present work is not directly concerned with animal metacognition, in order to engage in diagnostic reasoning to figure out how a system works, one must first recognize that more information is needed. Thus, we review evidence that non-human primates are able to recognize situations in which one’s own knowledge is incomplete or insufficient to achieve a goal or solve a cognitive problem. Based on this metacognitive awareness, one can diagnostically seek further information that is relevant for solving the task at hand.

In one study of animal information seeking, Call and Carpenter [28] varied whether chimpanzees and orangutans (*Pongo pygmaeus*) saw which of two opaque tubes had been baited with food. Apes were allowed to choose the contents from only one of the tubes; however, they were permitted to look inside the tubes before making their decision. Apes who did not see which tube had been baited with food were more likely to look inside the tubes than apes who saw the hiding location during the presentation, suggesting that apes can recognize when they need more information in order to achieve a goal (see also [29] for a similar result in monkeys). In another study, Beran and Smith [30] allowed rhesus (*Macaca mulatta*) and capuchin monkeys (*Cebus apella*) to seek additional information that would be relevant for solving a matching-to-sample task. Again, overall, both of these monkey species recognized when they needed more information in order to solve the task (although see [31]–[34] for some studies demonstrating that monkeys may be more limited in their metacognitive capacities).

Although both apes and monkeys have demonstrated the ability to recognize when they need more information to solve a cognitive task, all of the studies performed to date allowed participants to receive an immediate food reward for correct responses. Such immediate instrumental rewards may have motivated primate participants to engage in the information-seeking behaviors. Humans, in contrast, often seek out diagnostic information even in cases where no immediate reward is available. No work to date has explored whether primates (or any non-human animal) will engage in diagnostic behavior in the absence of a direct and immediate reward. In addition, despite growing evidence that several primate species engage in some information-seeking behaviors, there has been little work investigating whether primates will seek out missing information in the causal domain. Here, we attempt to explore whether primates are motivated to diagnose causal systems in the absence of an immediate reward.

Any study attempting to compare human and non-human causal information seeking, however, faces a bit of a methodological challenge. Most experimental tasks used to test causal understanding in primates (see review in [10]) have differed greatly from the tasks used to study children’s causal cognition. The use of similar methods across comparative and developmental participants has proven especially useful in other domains in which researchers have attempted to study similar questions across the two populations (see review in [35]). In the current paper, we try to overcome this issue of divergent methods by adapting a method commonly used in developmental psychology to study causal cognition in a non-human animal. Specifically, we adapted the blicket detector method of Gopnik and colleagues [1], [36] to investigate whether one non-human species– the brown capuchin monkey (*Cebus apella*)– will diagnose causal relations in the absence of an immediate reward. Although the blicket detector method was originally used to study whether preschool-aged children can discriminate between various patterns of statistical evidence to infer causal relationships, this method has already been adapted to study diagnostic causal learning in children [9] as well as causal learning in non-verbal populations (e.g., preverbal infants [37]). Here we apply this method for the first time to test a non-human primate population. We chose to test capuchin monkeys specifically because this species is a common non-human primate model of human cognition (see review in [38]) in part because of their rich social relationships, skilled tool use, and capacity for manipulating objects.

In the original blicket-detector studies, Gopnik and colleagues explored whether two- to four-year-old children could infer which objects had a novel causal power on the basis of different patterns of statistical evidence [1], [36]. In these studies, researchers presented children with a novel machine called a “blicket detector” and told children that “blickets make the machine go.” Across a number of studies, children made inferences about which objects were “blickets” based on whether the machine lit up, even though they were given relatively limited evidence about the kinds of objects that were able to activate the machine.

In one study, Gopnik et al. [1] presented children with two novel objects (A and B) that could potentially be “blickets.” In one condition, children observed a “one-cause” sequence that proceeded as follows: object A activated the machine by itself, object B did not activate the machine by itself, and then objects A and B activated the machine together twice. After witnessing this sequence, children reported that object A, but not object B, was a blicket (i.e., had causal efficacy). In another condition, children witnessed a similar but slightly different sequence involving two causes: object A activated the machine by itself three times, object B did not activate the machine by itself once, but then did activate the machine by itself twice. Here, children gave a different answer; they said that *both* objects A and B were blickets. Even though both of these testing conditions showed object B activating the machine with the same frequency (two out of three times), child participants distinguished between these conditions, suggesting that children may be using conditional probability information to determine which objects caused the machine to go.

In Experiment 1, we introduced capuchins to a “blicket detector” machine and validated this new method by testing the monkeys’ ability to perform simple discriminations. After introducing them to the detector, Experiments 2 and 3 presented capuchins with modified versions of Gopnik et al.’s [1] one-cause and two-cause conditions. As such, we were able to directly compare capuchins’ performance on this task with that of human children and see whether capuchins, like human children, think different objects activate the detector across the one-cause and two-cause conditions.

After establishing in Experiments 1–3 that monkeys understood the blicket detector paradigm, were comfortable interacting with the objects and the detector, and were sensitive to the distinction between the one-cause and two-cause conditions, we then performed Experiments 4 and 5 to investigate monkeys’ motivation to search for causal information about the blicket detector system. As reviewed above, one seemingly distinctive characteristic of human causal cognition is the “drive to explain” causal phenomena even when there are no direct or immediate benefits to acquiring such knowledge [39]. To test for this motivation in capuchins, Experiments 4 and 5 explored whether monkeys would spontaneously place objects on the blicket detector simply to learn which objects activate the machine even if there was no immediate opportunity to obtain a food reward. Do monkeys engage in a diagnostic search for information that might tell them about an underlying causal relationship, or do they instead focus only on acquiring causal knowledge in cases in which they need this information to achieve a direct outcome? An overview of the experiments is shown in Table 1.

**Table 1:** Overview of the experiments.

Exp. #	Research Question	Results
1	Can monkeys’ discriminate A+ vs. B− statistical evidence presentedin the blicket detector paradigm?	Yes. Monkeys chose object A (i.e., placed it on the blicket detector) more frequently than object B.
2	Can monkeys discriminate A+, B−, AB+ vs. C+, D−, D+ statistical evidence?(based on Gopnik et al., 2001)	Monkeys chose object A more frequently than object B, and object C more frequently than object D.
3	Can monkeys discriminate between the efficacy of objectsB and D from Exp. 2?	Yes. Monkeys chose object D more frequently than object B.
4	Will monkeys test novel objects to see if they produce an effect associatedwith the reward when no immediate food reward is available?	No. Monkeys generally did not spontaneously place objects on the detector when no immediate reward was available.
5	Will monkeys test novel objects to see if they produce an effect associatedwith the reward when no immediate food reward is available?(revised method)	Monkeys placed objects on the detector when prompted by the experimenter, but generally did not do so spontaneously. Monkeys did not seem to learn from evidence generated when the food reward was unavailable.

We began by introducing our participants to the blicket detector machine in order to find out whether monkeys could link any novel objects with the machine’s function. Experiment 1 began with this initial step, investigating whether capuchin monkeys could learn to distinguish between one object that always activated the blicket detector and another object that never activated the blicket detector.

## Experiment 1

### Methods

#### Animal care

This work was approved by the Yale University IACUC committee and conforms to federal guidelines for the use of animals in research. The capuchins live in a large social enclosure (4.1 m×3.2 m×2.5 m), which has multiple passageways between the sections that can be closed for separation of individuals or groups. The enclosure contains numerous toys and other monkeys for enrichment. The light cycle is a 12-hour cycle; the lights go on at 7 am and go off at 7 pm. The temperature is 72 degrees Fahrenheit (+/−2 degrees).

#### Participants

We tested six (three male: AG, JB, NN, three female: HG, JM, MD) brown capuchin monkeys (*Cebus apella*), a New World monkey species (see [38] for a more detailed account of capuchin ecology and social behavior). Our capuchin participants lived in a social enclosure at the Comparative Cognition Laboratory at Yale University (New Haven, CT). Monkeys received morning and afternoon feeds consisting of primate chow, vegetables, and fruit, which were supplemented by the food rewards they received for participating in experiments. Monkeys had ad libitum access to water. All monkeys had participated in a variety of other cognitive experiments, but none to date had tested their causal understanding.

#### Materials

**Figure 1:**
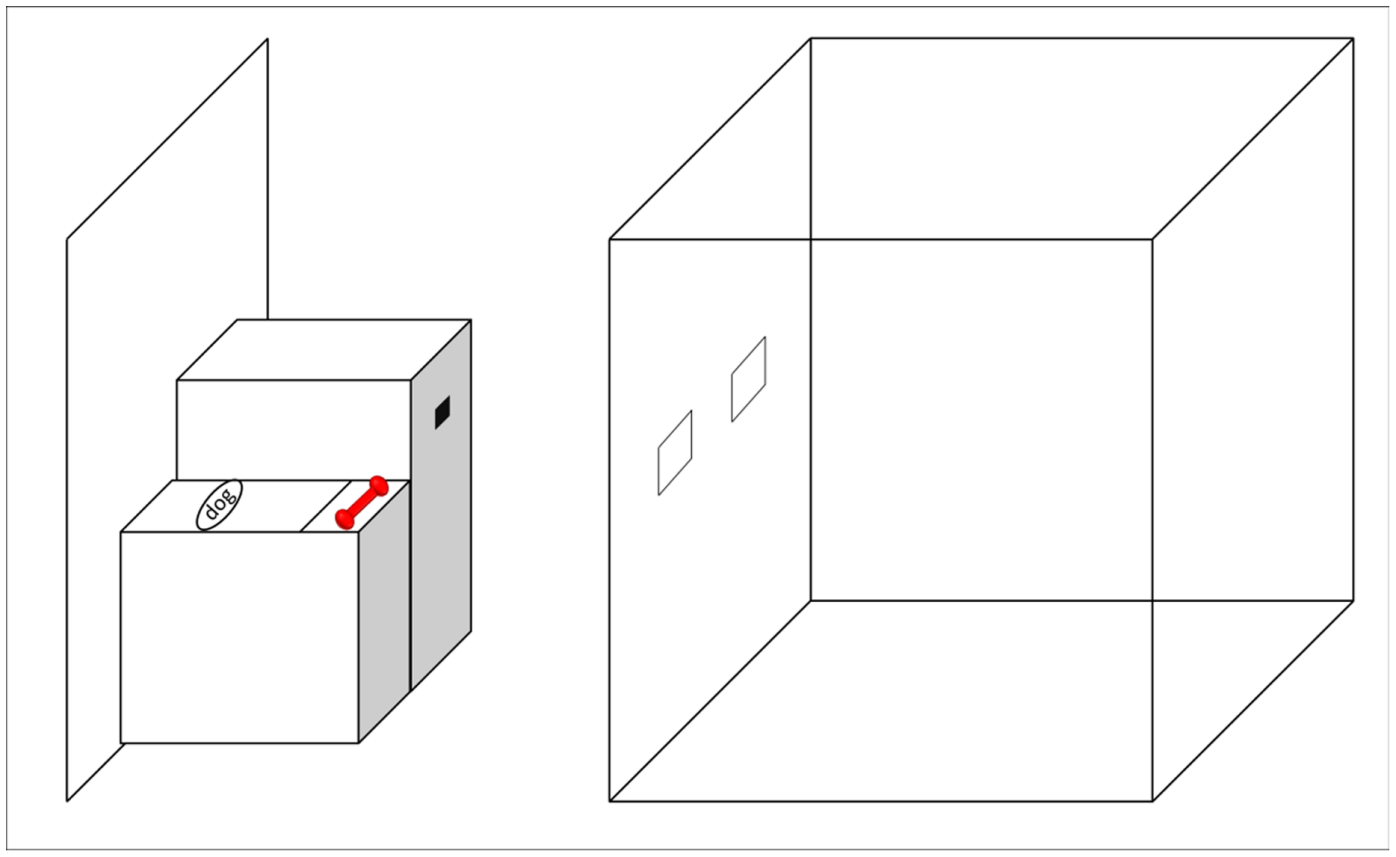
Apparatus used in Experiments 1–5. A depiction of the experimental setup used in Experiments 1–5, consisting of the blicket detector (left) and testing chamber (right). The blicket detector contained a platform for placing objects (location indicated by the red dumbbell), toy dog that lit up and made a sound when blickets were placed on the machine, and an inclined ramp “grape dispenser” that provided monkeys with a food reward when blickets were placed on the machine.

**Figure 2:**
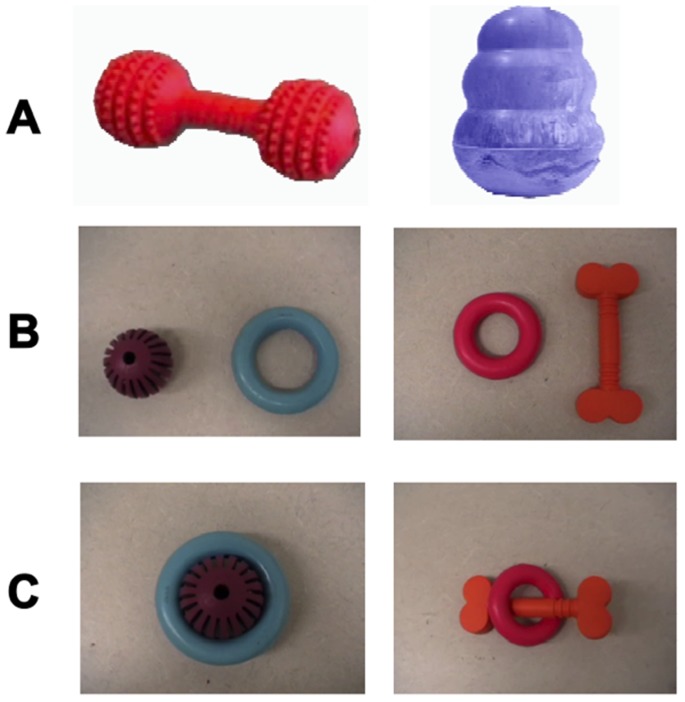
Objects used in Experiments 1–3. Experiment 1 (A) used a red dumbbell and blue Kong-toy, while Experiments 2 and 3 (B) used a blue ring and purple ball pair and an orange bone-shape and pink ring pair. Note that the pairs used in Experiments 2 and 3 could be linked together (C) such that they could be placed onto the detector as a unit.

We developed a “blicket detector” apparatus similar to the devices used by [1], [36] for testing human children. The experimental setup is shown in Figure 1. Our blicket detector was a foamcore box covered in black duct tape consisting of a 30 cm×35 cm×17 cm platform on which stimulus objects could be placed, which was connected to a larger 31 cm×21 cm×36 cm box. The larger box contained a 51 cm plastic ramp, which functioned as a grape dispenser. In contrast to the blicket detector used in children, our device delivered food rewards to the monkeys when they activated it. In addition to delivering food rewards, our device also gave a visual and auditory signal when activated. Specifically, a 12 cm×8 cm×6 cm battery-operated toy dog, which was located at the rear of the platform, lit up and made a squeaking sound when an experimenter activated it with a remote control. A 70 cm×66 cm barrier was placed behind the apparatus to allow an experimenter to surreptitiously operate the dog and grape dispenser. This experimenter surreptitiously observed the monkey through a 54 cm×5 cm slit in the barrier located 6 cm from the top. We used two small rubber toys as possible blickets: a 10 cm red dumbbell and a 7 cm blue cone-shaped “Kong toy” object (see Figure 2A). The red dumbbell always activated the machine while the blue cone never activated the machine. Although the monkeys previously had exposure to a variety of enrichment toys, they did not have previous experience with the specific objects used in the present experiments.

Monkeys participated in the experiment from inside a 75 cm×75 cm×75 cm cubic testing chamber located next to the blicket detector. The testing chamber was comprised of six wire-mesh panels. The panel on the side directly adjacent to the blicket detector contained two 9 cm×5 cm holes, which allowed monkeys to reach out of the testing chamber, place objects on the detector, and retrieve grapes from the bottom of the ramp. The testing chamber was isolated from the rest of the enclosure to avoid interference from other monkeys and prevent other monkeys from easily observing experimental sessions. In all experiments, monkeys willingly entered the testing area.

#### Procedure

The procedure consisted of three phases: a *shaping phase*, a *training phase*, and a *test phase.* In the *shaping phase*, we taught monkeys that the red dumbbell (the blicket) could be placed on the blicket detector and the monkeys were introduced to the detector’s features. Each shaping trial began when an experimenter placed the dumbbell inside the testing chamber. The experimenter held out his hand to encourage the monkey to hand the dumbbell back to him. Once the monkey put the blicket in the experimenter’s hand, the experimenter placed the blicket on the detector. When the dumbbell was placed on the detector, the detector began to “activate” – the mechanical dog lit up and began moving and a grape was dispensed down the incline. The monkey could not see that this activation was actually performed by a second experimenter hidden behind the detector who surreptitiously operated both the dog and grape dispenser. After approximately three seconds, the first experimenter removed the dumbbell from the platform, which seemed to shut off the activation of the detector. The temporal synchrony between the dumbbell’s placement on the platform, the dog’s activation, and the appearance of the grape was designed to give monkeys the impression that putting the dumbbell on the platform caused the machine’s activation. After approximately 20 trials in which the experimenter placed the dumbbell on the detector, the experimenter gradually started to prompt the monkey to put the dumbbell on the machine without help. Once a monkey began to place the dumbbell on the platform without help from the experimenter, the monkey advanced to the *training phase*.

In the training phase, we taught participants that different objects activated the detector at different rates. This phase consisted of two sessions of 10 training trials each. In five of the training trials in each session, the experimenter placed the red dumbbell (the blicket) inside the testing chamber. The monkey was then allowed to place this object on the blicket detector and when it did so, the hidden experimenter activated the machine. In the other five trials, however, the experimenter placed the blue cone (the non-blicket) inside the testing chamber. When the monkey put this object on the blicket detector, nothing happened. After three seconds, the experimenter removed this toy from the platform. In this way, the monkeys saw five trials in which the red dumbbell appeared to activate the machine, and five trials in which the blue cone appeared not to activate the machine. The order of the 10 trials was randomized subject to the constraint that each type of trial never appeared on more than two consecutive trials.

After two sessions of the training phase, subjects moved on to the *test phase*. The test phase consisted of a single session containing two familiarization trials followed by ten test trials. The familiarization trials were used to ensure that the monkeys remembered the statistical evidence presented during training; the familiarization trials were thus identical to the two trial types presented during the training phase, one involving the red dumbbell and one involving the blue cone. The order of the familiarization trials was chosen at random.

The goal of the test trials was to give the monkeys a choice between the two objects. In each test trial, the experimenter placed both the dumbbell and cone in the testing chamber. The position of the two objects (left versus right) alternated with every trial. The experimenter then allowed the monkey to enter the testing chamber. As in the training phase, if the monkey chose the dumbbell and placed it on the blicket detector, the hidden experimenter activated the machine. If the monkey placed the cone on the blicket detector, the hidden experimenter did not activate the machine. If a participant attempted to put a second object on the blicket detector, the first experimenter intercepted the second object before the monkey could place it on the machine. The monkey’s choice was coded as the first object it placed on the blicket detector.

AG had one previous test session that was excluded due to experimenter error in placing the objects inside the testing chamber, and MD had one previous test session that was aborted due to her disinterest.

### Results

The results of Experiment 1 are shown in Table 2. Monkeys placed the red dumbbell on the blicket detector more than the blue cone (Mean = 95% of trials). All six capuchins showed this preference (binomial probability: AG: *p*<0.002, HG: *p*<0.002, JB: *p*<0.01, JM: *p*<0.002, MD: *p = *0.057, NN: *p*<0.002). Five out of six monkeys (AG, HG, JM, MD, NN) chose the red dumbbell on the first test trial.

**Table 2:** Results of Experiments 1–3 across all participants.

Monkey	Experiment 1: # of TrialsChoosing Causally EffectiveObject	Experiment 2: One-CauseTask # of Trials ChoosingObject A	Experiment 2: Two-CauseTask # of Trials ChoosingObject C	Experiment 3: # of Trials Choosing Two-Cause Object D
AG	10/10	20/20	20/20	19/20
HG	10/10	–	–	–
JB	9/10	18/20	17/20	19/20
JM	10/10	20/20	20/20	19/20
MD	8/10	20/20	20/20	–
NN	10/10	20/20	20/20	20/20

### Discussion

Monkeys preferred the object that more frequently activated the blicket detector in the training phase. Indeed, all monkeys were able to learn which object activated the machine in our task with relatively minimal training. Thus, the results show that the blicket detector method can be successfully adapted for use with monkeys.

Having established a way to test monkeys’ preferences in the first experiment, our second experiment investigated whether capuchins could distinguish between two patterns of statistical evidence similar to those used in Gopnik et al.’s [1] study of children’s causal learning. We presented participants with a modified version of Gopnik et al.’s one-cause and two-cause conditions. In both conditions, one object always activated the machine by itself. In the two-cause condition, a second object sometimes activated the machine by itself, whereas in the one-cause condition, the corresponding object never activated the machine by itself. If monkeys are able to distinguish between these two conditions, we would expect them to show a greater tendency to choose the object that does not always activate the machine in the two-cause condition than in the one-cause condition.

## Experiment 2

### Methods

#### Participants

We tested five monkeys that participated in Experiment 1. One additional monkey (HG) began but could not fully complete testing because of a pregnancy and new baby.

#### Materials

We used the same blicket detector as in Experiment 1. The stimuli were four novel objects that were organized into two object-pairs (see Figures 2B and 2C). Each object in the pair could either be placed on the machine by itself or in a connected unit with its paired object. In this way, we required that monkeys sometimes place the two objects on the machine simultaneously. The first pair consisted of a 9 cm diameter blue ring (BR) and a 5 cm diameter purple ball (PB). The purple ball’s size was such that it could be fit snugly into the blue ring, thus enabling experimenters to present the stimuli either separated or “stuck” together. The second object pair was a 6 cm diameter pink ring (PR) and a 12 cm long orange bone (OB). Like the first object pair, these stimuli could be secured by forcing the bone through the pink ring, such that the ends of the bone prevented the stimuli from separating easily. We counterbalanced which object pair was used in each task and which object in each pair had which pattern of activation.

#### Procedure

Our procedure was modeled after the procedure used in Gopnik et al.’s [1] study of human children. Each monkey was tested on two different conditions: a *one-cause condition* and a *two-cause condition*. The order of these conditions was counterbalanced across monkeys. In both the one-cause and two-cause conditions, participants completed a *training phase* followed by two *test sessions*. Each training phase consisted of two cycles of three training sessions of 10 trials each. If there was a one-month or greater delay in between training sessions, monkeys received an additional cycle of three sessions to ensure that they retained the statistics presented earlier.

One-cause condition: On the first training session of the one-cause condition, monkeys saw that one object (A) alone activated the detector. In each trial, the experimenter placed object A inside the testing chamber. When monkeys placed this object on the blicket detector, the hidden experimenter activated the machine by operating the dog and rolling a grape down the incline. In the second training session, monkeys saw that a second object (B) did not activate the blicket detector when placed on the machine alone. In the third training session, monkeys saw that the connected unit of objects A and B together activated the detector.

Two-cause condition: The first two training sessions on the two-cause condition were identical to those of the one-cause condition: monkeys saw that object C alone activated the detector in the first session and that object D alone did not activate the blicket detector in the second session. In the third training session, however, monkeys were presented with object D alone which, when placed on the detector, did cause the hidden experimenter to activate the machine.

For both the one-cause and two-cause conditions, after completing the training phase, monkeys moved on to two sessions of the *test phase*. Each test session consisted of three familiarization trials to refresh the participant’s memory followed by 10 test trials. The three familiarization trials consisted of one trial of each of the three types of evidence presented during training. The procedure for the test trials was similar to that of Experiment 1; an experimenter placed both objects in the testing chamber and the monkey was allowed to choose one object to put on the machine. In the test trials for the one-cause (and two-cause) tasks, placing object A (C) on the detector activated the machine and placing object B (D) on the detector did not activate the machine.

### Results

The results of Experiment 2 are shown in Table 2. Monkeys chose object A over object B in the one-cause condition (Mean = 98% of trials) and object C over object D in the two-cause condition (Mean = 97% of trials). All five monkeys individually showed a statistical preference for object A in both the one-cause task (binomial probability: AG: *p*<0.0001, JB: *p*<0.0001, JM: *p*<0.0001, MD: *p*<0.0001, NN: *p*<0.0001) and object C in the two-cause task (binomial probability: AG: *p*<0.0001, JB: *p*<0.002, JM: *p*<0.0001, MD: *p*<0.0001, NN: *p*<0.0001). The data from each monkey’s first test trial are consistent with the overall trend. For the one-cause condition, all five monkeys chose object A on the first test trial, and for the two-cause condition, all five monkeys chose object C on the first test trial.

Out of all of these binomial tests in Experiments 1–3, all but one run showed no evidence for autocorrelation or was at ceiling, in which case autocorrelation cannot be assessed. However, JB made three incorrect choices in a row in the two-cause condition in Experiment 2, suggesting that his choices may have been autocorrelated, in which case a binomial test is not appropriate. There is not a commonly accepted generalization of the binomial test for autocorrelated data; we refer the reader directly to the data in the Supporting Information File S1 for visual inspection.

### Discussion

In both the one-cause and two-cause conditions, monkeys almost always chose to place object A and object C respectively on the blicket detector. Objects A and C were always associated with the blicket detector’s activation and objects B and D were associated with the blicket detector’s activation 50% of the time. In addition, we observed no differences across the two conditions; monkeys chose objects B and D equally across the one-cause and two-cause conditions. This pattern of performance differs from what was observed in human children [1]. Indeed, monkeys may have preferentially chosen object A and object C merely because these objects were more frequently associated with the machine’s activation and because monkeys always received a food reward for placing these objects on the machine.

At first glance, the data suggest that the monkeys did not discriminate between the one-cause and two-cause conditions. However, two considerations suggest that we should not necessarily interpret the results of Experiment 2 as evidence that capuchin monkeys are unable to distinguish between the two conditions. First, our non-verbal dependent measure of choice was somewhat different than the verbal dependent measure used by Gopnik et al. [1], in which children were asked to categorize objects as either blickets or non-blickets. In their study, children were allowed to say that both objects were blickets, whereas in our study, monkeys were forced to choose *only one* of the objects. Furthermore, in our task, it is likely that the monkeys were motivated to obtain as many grapes as possible. If participants were using this strategy, they should always choose objects A and C in the one-cause and two-cause conditions respectively even if they were sensitive to the difference between the conditions.

The second reason for caution involves an anecdotal observation we noted during the training phase of the one-cause condition. Interestingly, one monkey (JB) showed a surprising behavior on the training condition in which the connected unit of both objects A and B had to be placed on the detector together. On multiple occasions, JB spontaneously separated the two joined objects and tried to place only object A on the machine. This action provided at least an anecdotal suggestion that JB reasoned that *only* object A was causally responsible for the effect or that object B was an inhibitory cause even though he had been shown that the two objects together were associated with the machine’s activation. JB’s anecdotal novel intervention suggests a deeper understanding of the conditional probability information presented in the experiment.

We therefore performed a third experiment to determine whether the results in Experiment 2 should be attributed to monkeys’ inability to differentiate between the two conditions or whether an alternative aspect of the procedure masked monkeys’ capacity to perform this task. In Experiment 3, we presented monkeys with a choice between the one-cause condition object B and the two-cause condition object D. Both of these objects were correlated with the machine’s activation half of the time during familiarization trials; however, only the two-cause condition object D ever activated the machine by itself. The different patterns of evidence should lead to different inferences regarding each object’s causal efficacy. Thus, if monkeys are sensitive to the difference between the one-cause and two-cause cases, then they should prefer to place the two-cause condition object D on the blicket detector.

## Experiment 3

### Methods

#### Participants

We tested the five monkeys that completed Experiment 2 (AG, JB, JM, MD, NN). One monkey (MD) could not complete testing due to disinterest, as explained below.

#### Materials

The apparatus was the same blicket detector used in Experiments 1 and 2. The stimuli were the same four objects used in Experiment 2.

#### Procedure

Participants received three *training sessions* followed by two *test sessions*. Each training session consisted of three six-trial blocks involving the six types of evidence presented in the one-cause and two-cause conditions of Experiment 2. In each block, participants saw one trial of each of the three trial types used in the one-cause condition training (the one-cause object A activated the detector by itself, the one-cause object B did not activate the detector by itself, the one-cause objects A and B activated the detector together) and one trial of each of the three trial types used in the two-cause condition training (the two-cause object C activated the detector by itself, the two-cause object D did not activate the detector by itself, the two-cause object D activated the detector by itself). The order of the one-cause condition and two-cause condition sub-blocks was randomized. Within each sub-block, trials were presented in the order listed above except for the order of the two trials involving the two-cause object D, which was randomized.

The *test phase* of Experiment 3 consisted of 20 trials per participant, with a maximum of 10 trials per session. Each test session began with a six-trial familiarization that was identical to one of the six-trial blocks in the training sessions. Following the familiarization, participants received a maximum of 10 test trials in which they could choose between the one-cause object B and the two-cause object D. In the test trials, neither object activated the machine. Because monkeys were not reinforced in this session, we set up a criterion to be sure that they were motivated to continue performing the test trials. If a monkey did not place the object on the machine within 60 seconds, the experimenter allowed the participant to leave the testing chamber and attempted to redo the trial. If a trial was unsuccessfully attempted three times (i.e., the monkey did not place either object on the detector), the session was aborted and re-run on a different day. One monkey (MD) failed to complete testing even after multiple consecutive sessions and thus was dropped from the study.

### Results

The results of Experiment 3 are shown in Table 2. Overall, monkeys chose the two-cause object D more often than the one-cause object B (Mean = 96% of trials). Additionally, each monkey who successfully completed the testing showed a statistical preference for the two-cause object D (binomial probabilities: AG: *p*<0.0001, JB: *p*<0.0001, JM: *p*<0.0001, NN: *p*<0.0001). Additionally, on the first test trial, all four monkeys chose two-cause object D.

### Discussion

In a preference test between two objects that activated the blicket detector with equal frequency in the training phase, capuchins showed a strong preference for the object that activated the blicket detector conditionally independent of another object. These data show that capuchin monkeys make discriminations that are similar to those made by the children tested by Gopnik et al. [1]. Taken together, our results suggest that monkeys seem to treat our blicket task like a causal system, and thus our method can be useful for testing monkeys’ diagnostic abilities as well.

Because participants did not receive grapes during the test trial, they sometimes lost interest after putting the two-cause object D on the machine several times and not receiving a grape. Interestingly, rather than attempting to put the one-cause object B on the machine, the monkeys often preferred to place *neither object* on the machine, suggesting that they reasoned that putting the one-cause object B on the blicket detector would *never* cause the machine’s activation.

The monkeys’ refusal to place either object on the machine during the test trials suggests that their causal learning may be geared primarily, and perhaps exclusively, toward obtaining direct and immediate rewards. In Experiment 4, we decided to more directly investigate this potential difference in capuchins’ motivation to diagnose a novel causal structure by examining whether monkeys would place novel objects on a modified blicket detector device during times in which no food reward was available. If monkeys are curious about novel objects’ causal properties and motivated to diagnose such properties, then they should place novel objects on the blicket detector even if no immediate reward is present. However, if monkeys are only interested in using causal knowledge when doing so allows them to obtain food rewards, they should place objects on the blicket detector only when there is a possibility of gaining an immediate reward.

## Experiment 4

### Methods

#### Participants

We successfully tested four monkeys (two females and two males). One monkey (NN) had completed Experiments 1–3, one monkey (HG) had completed Experiment 1 and part of Experiment 2 but could not do Experiment 3 because of her recent baby, and two monkeys (FL and HR) were new to the task and had not completed any of the previous experiments in this study. We also began testing two other monkeys (AG and JM) that had completed Experiments 1–3, but these two monkeys were dropped before the end of Experiment 4 due to disinterest in entering the testing area.

#### Materials

We developed an updated version of the blicket detector apparatus used in Experiments 1–3. This new detector was coated in silver duct tape in order to distinguish it from the original machine and contained three foamcore sections. The first section measured 29 cm×42 cm×47 cm and contained a ramp angled down and away from the testing chamber at approximately 50 degrees. The second section consisted of a 20 cm×32 cm×47 cm stage atop which the same 12 cm×8 cm×6 cm battery-operated toy dog from Experiments 1–3 was positioned. As in Experiments 1–3, the dog lit up and emitted a squeaking sound when activated via a remote control. The third section, the grape dispenser, measured 29 cm×32 cm×47 cm and was attached by Velcro strips to the rest of the detector. The grape dispenser had a 4.5 cm×4 cm opening on both the front and the back, located 2.5 cm from the top and 11 cm from each side of both faces, through which grapes were dispensed using a small cup fixed to the end of a 48 cm wooden rod covered in green duct tape. As in Experiments 1–3, the machine was surreptitiously operated by an experimenter located behind a 76 cm×83 cm occluder attached to the back of the machine.

The objects used for testing included the two small rubber toys from Experiment 1 (the blicket red dumbbell and non-blicket blue Kong toy, hereafter referred to as object A and object B, respectively), as well as 15 pairs of novel objects consisting of small rubber or plastic toys. In each novel pair, one object always activated the blicket detector (referred to here as object C), and one object never activated the blicket detector (object D). Note that the novel objects used in this study are different from and therefore should not be confused with objects A–D from Experiments 2 and 3. The testing chamber was identical to the one used in previous experiments.

#### Procedure

The procedure consisted of four phases, which included three *training phases* followed by a *test phase*. The purpose of the training phases was to introduce the monkeys to the new blicket detector and the structure of the subsequent test trials. Monkeys typically received only one session per training day, although they were allowed to train more than once per day if they willingly re-entered the enclosure for an additional session.

In the *first training phase*, we familiarized our capuchin participants to the new blicket detector and allowed them to place familiar objects (A and B) on the detector. Although these objects were initially unfamiliar to monkeys who had not completed Experiment 1, those monkeys became familiar with these objects over the course of the Experiment 4 training. Each trial started when an experimenter placed objects A and B in the testing chamber, allowed the monkey to enter the chamber, and then encouraged the monkey to place one of the objects on the detector. If the monkey placed object A on the machine, the machine activated (i.e., the toy dog lit up and squeaked for approximately three seconds and the monkey also received a grape from the dispenser). If the monkey placed object B on the machine, nothing happened. After three seconds, the experimenter removed the object from the detector. The experimenter then returned the object to the monkey 20 seconds later.

In this first training phase, monkeys were given free access to both the detector and the grape dispenser. If a monkey failed to spontaneously place an object onto the blicket detector ramp in order to potentially activate the machine, an experimenter held out his hand to encourage the monkey to place the object onto the ramp. Each session continued until monkeys placed object A on the detector and activated it five times without solicitation from the experimenter. All monkeys completed a single initial session and one monkey (NN) also completed a second reminder session because more than two weeks passed between his first and second training sessions.

Monkeys then moved on to the *second training phase*. The goal of this second training phase was to teach the monkeys that they could receive a grape reward only when the grape dispenser was attached. The second training phase therefore consisted of timed trials in which the grape dispenser alternated between being attached and detached. In the trials in which the grape dispenser was attached, the machine activated and released a grape reward when a blicket was placed on the detector. In contrast, when the dispenser was detached, the machine did not deliver grapes when a blicket was placed on the detector; however, the dog still lit up and squeaked. Because the absence of a reward was more frustrating for the monkeys than we initially anticipated, we adjusted the length of each session depending on the monkeys’ perceived level of interest and motivation to continue testing. As such, in each testing session, participants received three to six trials of one to two and a half minutes each, depending on the monkeys’ perceived level of interest. During each session, half of the trials had the grape dispenser attached, and half had the dispenser detached. Each trial started when an experimenter placed both objects A and B in the testing chamber and allowed the monkey to enter the chamber and place the objects on the detector. Monkeys were then given free access to the two objects (objects A and B) and could place them on the detector without solicitation from the experimenter. As in the first phase, objects placed on the detector were returned to the monkey through a hole in the side of the testing chamber after 20 seconds. Between each trial, the monkey was allowed to leave the testing chamber and the dispenser was reset (i.e., the dispenser was either attached if it had been off, or detached if it had been on). To pass the second training phase, each monkey had to learn not to place objects on the detector when the dispenser was detached; monkeys advanced to the third training phase when they placed objects on the machine in a maximum of 25% of trials in which the grape dispenser was detached. Since the monkeys were already familiar with the causal properties of objects A and B, there was no value in placing these objects on the detector in the trials in which grapes were unavailable.

The *third training phase* was similar to the second phase except that (1) the period in which the grape dispenser was detached was shortened to 30 seconds and (2) monkeys were allowed to place only one of the two objects (A or B) on the detector during the period in which the grape dispenser was attached. The purpose of this training phase was to familiarize the monkeys with the procedure that would be used later in the test phase, most notably that they would be allowed only a *single choice* between the two objects during the period in which the dispenser was attached. Each session consisted of five trials. In order to move on to the test phase, monkeys needed to withhold placing the objects on the detector during the detached phase in at least three trials of a five-trial session. The number of sessions each monkey needed to advance to the test phase varied across monkeys (FL: three sessions; HR: one session; HG: one session and one additional reminder session because two weeks had passed between testing; NN: one session and one additional reminder session because two weeks had passed between testing). Additionally, one additional session with NN was begun but aborted and excluded from the final analysis due to a counterbalancing error.

The goal of the final *test phase* was to see if monkeys would spontaneously explore the causal properties of two novel objects– object C and object D– in the absence of a reward. As in the third training phase, each trial within the test sessions consisted of two parts: a 30-second period of free access to the blicket detector during which time the grape dispenser was detached followed by a single-choice trial in which the grape dispenser was attached. Note that even though monkeys could not receive a grape when the dispenser was detached, they could use these trials to place the novel objects on the detector and use the information about whether or not the dog activated to find out which object produced an effect associated with the reward. Thus, strategically testing the novel objects during this period could provide monkeys with potentially useful information that they could exploit during the subsequent single-choice trial.

Each test session consisted of 10 trials: five trials presented monkeys with the pair of familiar objects (A and B), while the other five involved novel object pairs. The purpose of the familiar object (A and B) trials was twofold. First, the inclusion of these familiar object trials evaluated whether monkeys would continue to respond correctly to known objects during the choice phase. Second, the known object trials were included to verify whether the monkeys remembered that they would not receive grapes when the grape dispenser was detached. The position of the objects, left or right, was randomized across trials. In addition, the object designated to be object C was counterbalanced across monkeys. Each monkey received three test sessions. If more than two weeks passed between test days, monkeys were presented with one additional session from the third training phase to remind them of the experiment’s structure before returning to the test sessions.

### Results

Four monkeys completed all the training phases. We were thus able to analyze how these monkeys performed during the test phase. We first examined whether monkeys used the 30-second period in which the dispenser was detached to strategically test the novel objects between which they could choose later in the single-choice trial. We observed surprisingly little strategic testing when the grape dispenser was detached. Only two of the four monkeys (FL and HG) ever placed a novel object on the blicket detector during the initial 30-second period in which the grape dispenser was detached. The two monkeys who did put novel objects on the blicket detector during the 30-second period did so on only 2 of 15 trials each. On FL’s third day of testing, he tried out object C and placed it on the machine during the detached-dispenser phase; during the single-choice phase, he correctly picked this object. In a later trial on the same day, FL also tried out a different object C, and then again chose correctly during the single-choice trial. On HG’s first trial on the first day of testing, she tried out object C, but then picked incorrectly in the single-choice trial. On HG’s first trial of the second day of testing, she tried out a different object C and then correctly chose this object in the single-choice trial. The results are shown in Table 3.

**Table 3:** Results of Experiments 4 and 5 across all participants.

Monkey	Experiment 4: # of Trials Placinga Novel Object on Machine	Experiment 4: # of Trials Choosing Object C	Experiment 5: # of Trials Placinga Novel Object on Machine	Experiment 5: # of Trials Choosing Object C
FL	2/15	2/2	2/5	1/2
HG	2/15	1/2	0/5	–
HR	0/15	–	0/5	–
NN	0/15	–	0/5	–

Despite the fact that monkeys rarely placed the novel objects on the detector, they did perform relatively accurately with familiar objects A and B. During the choice phase, all four monkeys correctly chose object A 100% of the time. Moreover, monkeys only rarely placed the familiar objects on the detector during the 30-second period in which the dispenser was detached (NN and HR never placed either object A or B, FL placed B once, and HG placed A four times throughout testing with three times occurring within a single 30-second block). This behavior was expected because testing the familiar objects A and B would not provide any new information.

### Discussion

The goal of Experiment 4 was to examine if monkeys would strategically test objects on the blicket detector during a time in which they could not receive a food reward. Even in a situation in which strategic testing might benefit the monkeys, we observed little testing. Only two of four monkeys ever tried to use the detector in the absence of a reward, and even they did so only on two trials each. Monkeys’ failure to place objects on the blicket detector during the “grape dispenser detached” phase suggests that monkeys may be uninterested in making causal diagnoses in the absence of immediate instrumental rewards. Interestingly, this pattern of behavior persisted even though monkeys repeatedly observed that the grape dispenser would be reattached in subsequent trials. It therefore seems that monkeys may be willing to use the blicket detector to exploit immediately available rewards, but that they are not interested in using the detector to learn about causal relations in the absence of those rewards.

Because the monkeys so rarely tested the novel objects in the dispenser- detached phase, we could not examine whether they are able to learn about the effectiveness of objects without an immediate reward and use that knowledge at a later time. In Experiment 5 we attempted to stimulate the monkeys’ interest in testing the novel objects to see if the objects are causally effective despite no possibility for an immediate reward.

## Experiment 5

### Methods

#### Participants

We tested the four monkeys who completed Experiment 4 (FL, HG, HR, and NN).

#### Materials

We used the same blicket detector as in Experiment 4. The stimuli were 10 more pairs of novel objects similar to the stimuli used in Experiment 4.

#### Procedure

Monkeys received one *training session* and one *test session*. The training session consisted of 10 trials: five trials presented monkeys with the pair of familiar objects (A and B), while the other five involved novel object pairs. The format of these trials was identical to that of the test session of Experiment 4 (i.e., there was a period in which the grape dispenser was detached during which the monkeys could test the objects followed by a single-choice trial with the dispenser reattached) except that during the period in which the grape dispenser was detached, the experimenter solicited the monkey to put the objects on the blicket detector. Although most monkeys responded to this solicitation, some monkeys still refused to place objects on the machine when the dispenser was detached. If a monkey required more than two and a half minutes of solicitation to put the objects on the detector in a given trial, we then switched to a different form of training in which the experimenter demonstrated the objects’ properties for the monkeys by placing the novel objects on the detector.

Once monkeys completed a single training session with prompting (or demonstration if necessary), they received one test session. This single test session was identical to the test sessions used in Experiment 4; each monkey received five trials with the pair of familiar objects (A and B) and five trials with the novel object pairs (C and D), but no solicitation or demonstration was provided.

### Results

In the training session of Experiment 5, all four monkeys placed the novel objects on the blicket detector after being solicited to do so by the experimenter on at least some of the trials (HR: 5/5, NN: 4/5, HG: 4/5, FL: 2/5). The monkeys, however, did not seem to learn from the evidence they acquired in the subsequent single-choice trials when the grape dispenser was attached. In the corresponding single-choice trials that followed the periods in which monkeys successfully tried out the novel objects, none of the four monkeys exhibited a significant preference for placing the blicket on the machine (HR: 3/5, NN: 3/4, HG: 2/4, FL: 0/2). Monkeys did, however, continue to perform accurately with familiar objects A and B in the training session of Experiment 5. All monkeys chose object A 100% of the time during the choice phase. In addition, monkeys rarely tried out either object A or B when the dispenser was detached (FL, HG, and HR never placed A or B when the dispenser was detached and NN placed object A once).

Monkeys’ performance with novel objects in the test session that followed (in which there was no object solicitation) matched their poor performance in the previous training session. Only one of the monkeys (FL) placed either novel object on the detector when the grape dispenser was detached (FL: 2/5, HR: 0/5, NN: 0/5, HG: 0/5). Both of these times, FL tested only the non-blicket (object D) during the dispenser-detached period. In the subsequent choice phase, he correctly picked the blicket (object C) on one of two trials. Despite this poor performance with novel objects, monkeys continued to perform well with objects A and B; on the familiar object trials, monkeys never placed A or B on the machine when the dispenser was detached and they always chose object A during the choice phase. The results are shown in Table 3.

### Discussion

In Experiment 5, monkeys tended to gather evidence about which objects were blickets only when actively prompted by the experimenter to place objects on the machine. However, even after observing this information, monkeys did not reliably use this evidence to help them decide which object to choose in the choice phase. Indeed, it seems that, even when prompted to use the detector strategically, monkeys failed to use this training to learn about the causal properties of novel objects in the absence of a reward.

This pattern of results suggests a sharp contrast between the motivation underlying human and capuchin causal learning. Even young human children regularly explore causal systems to learn how the systems work even when there are no direct benefits to obtaining such knowledge. For example, as reviewed earlier, Schulz and Bonawitz [6] found that children spontaneously disambiguated causally confounded evidence regarding how a toy worked. Capuchins, in contrast, showed no interest in the task whenever they were unable to immediately obtain food. Indeed, capuchins chose not to test potential blickets even when this knowledge might prove useful just a few seconds later. Additionally, monkeys did not succeed at using the evidence they acquired in the “grape-dispenser detached” phase when making decisions about which object to put on the blicket detector in the single-choice trials. Human children, however, make accurate causal inferences even when presented with very limited evidence, sometimes even a single trial. Although it is not clear whether monkeys had difficulty in the single-choice trials because they (1) needed a greater amount of evidence to infer causal patterns or (2) did not encode the information they received when the grape dispenser was detached, the results of this experiment suggest potentially critical differences between the nature of human and capuchin causal learning.

## General Discussion

The experiments presented here attempted to explore whether one signature of human causal cognition– the motivation to search diagnostically for causal knowledge– is present in a non-human-primate species, the brown capuchin monkey. Specifically, we investigated whether monkeys would be willing to test out novel objects in the absence of an immediate food reward. Human causal learning often involves just this form of diagnosis; it is geared towards learning for the sake of learning. Capuchin monkeys, however, were uninterested in diagnosing which objects activated a machine in the absence of a reward. Although monkeys regularly exploited this causal system in Experiments 1–3 when they could obtain a food reward, monkeys stopped placing objects on the blicket detector when no immediate food reward was available in Experiments 4 and 5, even when such interventions were clearly diagnostic for the trial that would follow. These results suggest that capuchin causal cognition may be exploitative, rather than exploratory in nature.

Experiment 5 also revealed that monkeys did not seem to learn from the statistical evidence they received when the grape dispenser was detached from the blicket detector. In Experiment 5, monkeys performed at chance in the choice trials even when they had previously tested one of the two objects. Unlike our monkey subjects, people routinely infer causal structures and make accurate inductive inferences from limited evidence (see [40] for a review). Not only were the monkeys more likely to interact with the machine when a food reward was available, they were also better at learning from the evidence in those trials.

These results suggest an important limitation on animals’ ability to seek information about the causal world; they may do so only when there is a direct benefit. Previous work has demonstrated that several non-human primate species, including capuchins, spontaneously seek potentially useful information when a direct and immediate food reward is available (e.g., [28]–[30]). Our monkeys’ successful use of the blicket detector in Experiments 1–3 is consistent with this pattern of behavior.

However, once we removed the opportunity for capuchins to obtain an immediate food reward in Experiments 4 and 5, monkeys no longer tested novel objects on the blicket detector. As such, capuchin monkeys’ information-seeking behavior appears to be motivated solely by the prospect of obtaining a food reward. In contrast to children’s exploratory play and other information-seeking behaviors, capuchin monkeys’ (and perhaps other primates’) information seeking does not seem to be aimed at learning how the object works so much as learning how to obtain a reward. In this respect, our data are consistent with associative learning theories, which propose that in the absence of rewards associated with specific behaviors, animals explore their environment randomly as opposed to diagnostically.

It is worth noting, however, that the present studies tested only one primate species on a single experimental task, and that the sample sizes were relatively small. Thus, future work might explore whether other primate species would be more likely to seek information about causal relations in the absence of an immediate reward. Additionally, we can ask whether capuchins or other primates would be more likely to engage in diagnostic behavior when learning about a more ecologically relevant system.

In one study of chimpanzee play across various reward conditions, Clark and Smith [41] presented chimpanzees with a maze-like cognitive challenge device in which chimpanzees could use tools to move a food or non-food reward through the maze. Surprisingly, chimpanzees played with the device more frequently in trials in which a non-food reward (a token) was available than in trials in which a food reward (Brazil nuts) was available. Clark and Smith suggested that chimpanzees played with the device because it was intrinsically interesting (see also [42]). However, this study differed substantially from our experiments in that Clark and Smith’s task did not involve learning causal relations or information seeking. Furthermore, there is no evidence that chimpanzees in Clark and Smith’s study interacted with the device in a diagnostic manner.

Comparative researchers have typically attempted to explain the gap between human and animal causal cognition by suggesting that some causal learning mechanisms are present in humans, but not in other animals (e.g., higher-order inferential reasoning or learning from conditional probability statistics, see [10], [11]). Our finding that capuchins lack the tendency to spontaneously diagnose causal structures and learn from evidence in situations in which no immediate reward is available suggests that differences in competence may not be the whole story. Our results suggest that the gap between human and animal causal cognition is not merely a gap in *competence*, but is perhaps also a gap in *motivation*.

## Supporting Information

File S1 **Supporting information contains the trial-by-trial data for each monkey for Experiments 1–3.**
(DOCX)

## References

[1] Gopnik A, Sobel DM, Schulz LE, Glymour C (2001) Causal learning mechanisms in very young children: Two-, three-, and four-year-olds infer causal relations from patterns of variation and covariation. Dev Psychol 37: 620–629. 11552758

[2] Lombrozo T (2006) The structure and function of explanations. Trends Cogn Sci 10: 464–470. 16942895 10.1016/j.tics.2006.08.004

[3] Lombrozo T (2012) Explanation and abductive inference. In: Holyoak KJ, Morrison RG, editors. Oxford handbook of thinking and reasoning. Oxford, UK: Oxford University Press. 270–276.

[4] Gopnik A, Glymour C, Sobel DM, Schulz LE, Kushnir T, et al (2004) A theory of causal learning in children: Causal maps and Bayes nets. Psychol Rev 111: 1–30. 10.1037/0033-295X.111.1.3 14756583

[5] Gopnik A, Schulz LE (2004) Mechanisms of theory formation in young children. Trends Cogn Sci 8: 371–377. 15335464 10.1016/j.tics.2004.06.005

[6] Schulz LE, Bonawitz EB (2007) Serious fun: Preschoolers engage in more exploratory play when evidence is confounded. Dev Psychol 43: 1045–1050. 17605535 10.1037/0012-1649.43.4.1045

[7] Cook C, Goodman N, Schulz LE (2011) Where science starts: Spontaneous experiments in preschoolers’ exploratory play. Cognition 120: 341–349. 21561605 10.1016/j.cognition.2011.03.003

[8] Gweon H, Schulz L (2008) Stretching to learn: Ambiguous evidence and variability in preschoolers’ exploratory play. In: Love BC, McRae K, Sloutsky VM, editors. Proceedings of the 30^th^ Annual Conference of the Cognitive Science Society. Washington, DC: Cognitive Science Society.

[9] Legare CH (2012) Exploring explanation: Explaining inconsistent evidence informs exploratory, hypothesis-testing behavior in young children. Child Dev 83: 173–185. 22172010 10.1111/j.1467-8624.2011.01691.x

[10] Penn DC, Holyoak KJ, Povinelli DJ (2008) Darwin’s mistake: Explaining the discontinuity between human and nonhuman minds. Behav Brain Sci 31: 109–178. 18479531 10.1017/S0140525X08003543

[11] Penn DC, Povinelli DJ (2007) Causal cognition in human and nonhuman animals: A comparative, critical review. Annu Rev Psychol 58: 97–118. 17029564 10.1146/annurev.psych.58.110405.085555

[12] Rescorla RA, Wagner AR (1972) A theory of Pavlovian conditioning: Variations in the effectiveness of reinforcement and nonreinforcement. In: Black AH, Prokasy WF, editors. Classical conditioning II: Current research and theory. New York: Appleton-Century-Crofts. 64–99.

[13] Dickinson A (2001) Causal learning: An associative analysis. Q J Exp Psychol 54: 3–25. 10.1080/02724990042000010 11216300

[14] Pearce JM, Bouton ME (2001) Theories of associative learning in animals. Annu Rev Psychol 52: 111–139. 11148301 10.1146/annurev.psych.52.1.111

[15] Shanks DR (1995) The psychology of associative learning. London: Cambridge University Press.

[16] Wasserman EA, Miller RR (1997) What’s elementary about associative learning? Annu Rev Psychol 48: 573–607. 9046569 10.1146/annurev.psych.48.1.573

[17] Blaisdell AP, Sawa K, Leising KJ, Waldmann MR (2006) Causal reasoning in rats. Science 311: 1020–1022. 16484500 10.1126/science.1121872

[18] Waldmann MR, Hagmayer Y (2005) Seeing vs. doing: Two modes of accessing causal knowledge. J Exp Psychol Learn Mem Cogn 31: 216–227. 15755240 10.1037/0278-7393.31.2.216

[19] Kutlu MG, Schmajuk NA (2012) Classical conditioning mechanisms can differentiate between seeing and doing in rats. J Exp Psychol Anim Behav Process 38: 84–101. 22229589 10.1037/a0026221

[20] Skinner BF (1957) Verbal behavior. New York: Appleton-Century-Crofts.

[21] Povinelli DJ, Dunphy-Lelii S (2001) Do chimpanzees seek explanations? Preliminary comparative investigations. Can J Exp Psychol 55: 185–193. 11433789 10.1037/h0087365

[22] Hayashi M (2007) Stacking of blocks by chimpanzees: Developmental processes and physical understanding. Anim Cogn 10: 89–103. 16909233 10.1007/s10071-006-0040-9

[23] Hayashi M, Takeshita H (2009) Stacking of irregularly shaped blocks in chimpanzees (*Pan troglodytes*) and young humans (*Homo sapiens*). Anim Cogn 12: S49–S58. 19672637 10.1007/s10071-009-0273-5

[24] Brauer J, Kaminski J, Riedel J, Call J, Tomasello M (2006) Making inferences about the location of hidden food: Social dog, causal ape. J Comp Psychol 120: 38–47. 16551163 10.1037/0735-7036.120.1.38

[25] Call J (2004) Inferences about the location of food in the great apes (*Pan paniscus*, *Pan troglodytes*, *Gorilla gorilla*, and *Pongo pygmaeus*). J Comp Psychol 118: 232–240. 15250810 10.1037/0735-7036.118.2.232

[26] Hanus D, Call J (2008) Chimpanzees infer the location of a reward on the basis of the effect of its weight. Curr Biol 18: R370–R372. 18460313 10.1016/j.cub.2008.02.039

[27] Smith JD, Shields WE, Washburn DA (2003) A comparative approach to metacognition and uncertainty monitoring. Behav Brain Sci 26: 317–339. 14968691 10.1017/s0140525x03000086

[28] Call J, Carpenter M (2001) Do apes and children know what they have seen? Anim Cogn 4: 207–220.

[29] Hampton RR, Zivin A, Murray EA (2004) Rhesus monkeys (*Macaca mulatta*) discriminate between knowing and not knowing and collect information as needed before acting. Anim Cogn 7: 239–246. 15105996 10.1007/s10071-004-0215-1

[30] Beran MJ, Smith JD (2011) Information seeking by rhesus monkeys (*Macaca mulatta*) and capuchin monkeys (*Cebus apella*). Cognition 120: 90–105. 21459372 10.1016/j.cognition.2011.02.016 PMC3095768

[31] Basile BM, Hampton RR, Suomi SJ, Murray EA (2009) An assessment of memory awareness in tufted capuchin monkeys (*Cebus apella*). Anim Cogn 12: 169–180. 18712532 10.1007/s10071-008-0180-1 PMC2676690

[32] Beran MJ, Smith JD, Coutinho MV, Couchman JJ, Boomer J (2009) The psychological organization of “uncertainty” responses and “middle” responses: A dissociation in capuchin monkeys (*Cebus apella*). J Exp Psychol Anim Behav Process 35: 371–381. 19594282 10.1037/a0014626 PMC3901429

[33] Fujita K (2009) Metamemory in tufted capuchin monkeys (*Cebus apella*). Anim Cogn 12: 575–585. 19242741 10.1007/s10071-009-0217-0

[34] Paukner A, Anderson JR, Fujita K (2006) Redundant food searches by capuchin monkeys (*Cebus apella*): A failure of metacognition? Anim Cogn 9: 110–117. 16184375 10.1007/s10071-005-0007-2

[35] Kinzler KD, Spelke ES (2007) Core systems in human cognition. Prog Brain Res 164: 257–264. 17920436 10.1016/S0079-6123(07)64014-X

[36] Gopnik A, Sobel DM (2000) Detecting blickets: How young children use information about novel causal powers in categorization and induction. Child Dev 71: 1205–1222. 11108092 10.1111/1467-8624.00224

[37] Sobel DM, Kirkham NZ (2007) Babies and blickets: The development of causal reasoning in toddlers and infants. Dev Sci 10: 298–306. 17087545 10.1037/0012-1649.42.6.1103

[38] Fragaszy DM, Visalberghi E, Fedigan LM (2004) The complete capuchin. Cambridge, England: Cambridge University Press.

[39] Gopnik A (2000) Explanation as orgasm and the drive for causal understanding. In: Keil FC, Wilson RA, editors. Explanation and cognition. Cambridge, MA: MIT Press. 299–322.

[40] Tenenbaum JB, Griffiths TL, Kemp C (2006) Theory-based Bayesian models of inductive learning and reasoning. Trends Cogn Sci 10: 309–318. 16797219 10.1016/j.tics.2006.05.009

[41] Clark FE, Smith LJ (2013) Effect of a cognitive challenge device containing food and non-food rewards on chimpanzee well-being. Am J Primatol 75: 807–816. 23436455 10.1002/ajp.22141

[42] Harlow HF (1950) Learning and satiation of response in intrinsically motivated complex puzzle performance by monkeys. J Comp Physiol Psychol 43: 289–294. 15436888 10.1037/h0058114

