# Supplementary material for: Do Capuchin Monkeys (Cebus apella) Diagnose Causal Relations in the Absence of a Direct Reward?
Source: PLoS One. 2014 Feb 19;9(2):e88595. doi: 10.1371/journal.pone.0088595 (PMC3929502; doi:10.1371/journal.pone.0088595)
Supplement: File S1 — Supporting information contains the trial-by-trial data for each monkey for Experiments 1–3. (DOCX) [file pone.0088595.s001.docx]

**Trial-by-Trial Data for “Do Capuchin Monkeys (*Cebus apella*) Diagnose Causal Relations in the Absence of a Direct Reward?”**

This supporting information contains the trial-by-trial data for each monkey for Experiments 1-3.

**Table S1. Experiment 1 Results.**

| **Trial** | **AG** | **HG** | **JB** | **JM** | **MD** | **NN** |
| --- | --- | --- | --- | --- | --- | --- |
| Trial 1 | 1 | 1 | 0 | 1 | 1 | 1 |
| Trial 2 | 1 | 1 | 1 | 1 | 1 | 1 |
| Trial 3 | 1 | 1 | 1 | 1 | 1 | 1 |
| Trial 4 | 1 | 1 | 1 | 1 | 1 | 1 |
| Trial 5 | 1 | 1 | 1 | 1 | 0 | 1 |
| Trial 6 | 1 | 1 | 1 | 1 | 1 | 1 |
| Trial 7 | 1 | 1 | 1 | 1 | 1 | 1 |
| Trial 8 | 1 | 1 | 1 | 1 | 1 | 1 |
| Trial 9 | 1 | 1 | 1 | 1 | 1 | 1 |
| Trial 10 | 1 | 1 | 1 | 1 | 0 | 1 |

1 = Monkey chose object A (correct), 0 = Monkey chose object B (incorrect).

**Table S2. Experiment 2 Results: One-Cause Condition.**

| **Trial** | **AG** | **JB** | **JM** | **MD** | **NN** |
| --- | --- | --- | --- | --- | --- |
| Day 1 Trial 1 | 1 | 1 | 1 | 1 | 1 |
| Day 1 Trial 2 | 1 | 0 | 1 | 1 | 1 |
| Day 1 Trial 3 | 1 | 1 | 1 | 1 | 1 |
| Day 1 Trial 4 | 1 | 1 | 1 | 1 | 1 |
| Day 1 Trial 5 | 1 | 1 | 1 | 1 | 1 |
| Day 1 Trial 6 | 1 | 1 | 1 | 1 | 1 |
| Day 1 Trial 7 | 1 | 1 | 1 | 1 | 1 |
| Day 1 Trial 8 | 1 | 0 | 1 | 1 | 1 |
| Day 1 Trial 9 | 1 | 1 | 1 | 1 | 1 |
| Day 1 Trial 10 | 1 | 1 | 1 | 1 | 1 |
| Day 2 Trial 1 | 1 | 1 | 1 | 1 | 1 |
| Day 2 Trial 2 | 1 | 1 | 1 | 1 | 1 |
| Day 2 Trial 3 | 1 | 1 | 1 | 1 | 1 |
| Day 2 Trial 4 | 1 | 1 | 1 | 1 | 1 |
| Day 2 Trial 5 | 1 | 1 | 1 | 1 | 1 |
| Day 2 Trial 6 | 1 | 1 | 1 | 1 | 1 |
| Day 2 Trial 7 | 1 | 1 | 1 | 1 | 1 |
| Day 2 Trial 8 | 1 | 1 | 1 | 1 | 1 |
| Day 2 Trial 9 | 1 | 1 | 1 | 1 | 1 |
| Day 2 Trial 10 | 1 | 1 | 1 | 1 | 1 |

1 = Monkey chose object A (correct), 0 = Monkey chose object B (incorrect).

**Table S3. Experiment 2 Results: Two-Cause Condition.**

| **Trial** | **AG** | **JB** | **JM** | **MD** | **NN** |
| --- | --- | --- | --- | --- | --- |
| Day 1 Trial 1 | 1 | 1 | 1 | 1 | 1 |
| Day 1 Trial 2 | 1 | 1 | 1 | 1 | 1 |
| Day 1 Trial 3 | 1 | 1 | 1 | 1 | 1 |
| Day 1 Trial 4 | 1 | 1 | 1 | 1 | 1 |
| Day 1 Trial 5 | 1 | 1 | 1 | 1 | 1 |
| Day 1 Trial 6 | 1 | 0 | 1 | 1 | 1 |
| Day 1 Trial 7 | 1 | 0 | 1 | 1 | 1 |
| Day 1 Trial 8 | 1 | 0 | 1 | 1 | 1 |
| Day 1 Trial 9 | 1 | 1 | 1 | 1 | 1 |
| Day 1 Trial 10 | 1 | 1 | 1 | 1 | 1 |
| Day 2 Trial 1 | 1 | 1 | 1 | 1 | 1 |
| Day 2 Trial 2 | 1 | 1 | 1 | 1 | 1 |
| Day 2 Trial 3 | 1 | 1 | 1 | 1 | 1 |
| Day 2 Trial 4 | 1 | 1 | 1 | 1 | 1 |
| Day 2 Trial 5 | 1 | 1 | 1 | 1 | 1 |
| Day 2 Trial 6 | 1 | 1 | 1 | 1 | 1 |
| Day 2 Trial 7 | 1 | 1 | 1 | 1 | 1 |
| Day 2 Trial 8 | 1 | 1 | 1 | 1 | 1 |
| Day 2 Trial 9 | 1 | 1 | 1 | 1 | 1 |
| Day 2 Trial 10 | 1 | 1 | 1 | 1 | 1 |

1 = Monkey chose object C (correct), 0 = Monkey chose object D (incorrect).

**Table S4. Experiment 3 Results.**

| **Trial** | **AG** | **JB** | **JM** | **NN** |
| --- | --- | --- | --- | --- |
| Day 1 Trial 1 | 1 | 1 | 1 | 1 |
| Day 1 Trial 2 | 1 | 1 | 1 | 1 |
| Day 1 Trial 3 | 1 | 1 | 1 | 1 |
| Day 1 Trial 4 | 1 | 1 | 1 | 1 |
| Day 1 Trial 5 | 1 | 1 | 1 | 1 |
| Day 1 Trial 6 | 1 | 1 | 1 | 1 |
| Day 1 Trial 7 | 1 | 1 | 1 | 1 |
| Day 1 Trial 8 | X | 1 | 1 | 1 |
| Day 1 Trial 9 | X | 1 | 1 | 1 |
| Day 1 Trial 10 | X | 1 | 1 | 1 |
| Day 2 Trial 1 | 1 | 1 | 1 | 1 |
| Day 2 Trial 2 | 1 | 1 | 1 | 1 |
| Day 2 Trial 3 | 1 | 0 | 1 | 1 |
| Day 2 Trial 4 | 0 | 1 | 1 | 1 |
| Day 2 Trial 5 | 1 | 1 | 1 | 1 |
| Day 2 Trial 6 | 1 | 1 | 1 | 1 |
| Day 2 Trial 7 | 1 | 1 | 1 | 1 |
| Day 2 Trial 8 | 1 | 1 | 1 | 1 |
| Day 2 Trial 9 | 1 | 1 | 1 | 1 |
| Day 2 Trial 10 | 1 | 1 | 0 | 1 |
| Day 3 Trial 1 | 1 | X | X | X |
| Day 3 Trial 2 | 1 | X | X | X |
| Day 3 Trial 3 | 1 | X | X | X |

1 = Monkey chose object D (correct), 0 = Monkey chose object B (incorrect). Due to disinterest, AG only received seven test trials on day 1. Thus, AG received a third day of testing containing the remaining three test trials.
